# Degradation Efficiency and Mechanism Exploration of an Fe_78_Si_9_B_13_ Metallic Glass Cathode in the Electro-Fenton Degradation of p-NP

**DOI:** 10.3390/ma18050930

**Published:** 2025-02-20

**Authors:** Jiatao Xie, Shengkang Hu, Mengyuan Wei, Shenghui Xie

**Affiliations:** 1 School of Mechanical and Automotive Engineering, South China University of Technology, Guangzhou 510640, China; 15626232817@163.com; 2 Guangdong Provincial Key Laboratory of New Energy Materials Service Safety, Shenzhen Engineering Laboratory for Advanced Technology of Ceramic, Shenzhen Key Laboratory of Special Functional Materials, College of Materials Science and Engineering, Shenzhen University, Shenzhen 518060, China; 2200341028@email.szu.edu.cn

**Keywords:** Fe-based metallic glass, electro-Fenton system, heterogeneous catalysis, p-nitrophenol, hydroxyl radical

## Abstract

Fe-based metallic glass (MG) exhibits excellent performance as a heterogeneous catalyst in degradation but is rarely used as a working electrode in electro-Fenton (EF) systems. We used Fe_78_Si_9_B_13_ MG as the working electrode to investigate the effect of the EF process on the degradation efficiency of p-nitrophenol (p-NP). The EF system had the highest catalytic efficiency (the reaction rate was 3.4 times that of chemical degradation) at a voltage of −1 V (vs. SCE) and showed 95.6% degradation of p-NP within 30 min. The electrode voltage accelerated the generation of hydroxyl radicals (·OH) in the system, thus promoting pollutant degradation. In addition, the Fe_78_Si_9_B_13_ MG cathode demonstrated good structural stability and reusability after 10 cycles. Fe_78_Si_9_B_13_ MG ribbons can serve as a suitable cathode material and provide potential optimization solutions for the degradation of organic pollutants.

## 1. Introduction

The treatment of organic pollution has always been a thorny problem [1,2,3,4,5], and there are three common processing methods: physical adsorption, biodegradation, and advanced oxidation processes (AOPs). Among them, heterogeneous Fenton technology is an effective AOP that has significant effects in the treatment of refractory organic pollutants, but it also faces drawbacks such as the production of a large amount of iron sludge and high costs. Therefore, improved technologies such as optical Fenton, electro-Fenton, and magnetic-Fenton have been derived [6,7,8,9,10,11]. Among them, the electro-Fenton (EF) process not only generates H_2_O_2_ in situ at the cathode, solving the storage and transportation problems of hydrogen peroxide (H_2_O_2_), but also reduces Fe^3+^ to Fe^2+^ at the cathode in the EF system, improving the utilization rate of the raw material. The degradation efficiency of organic pollutants related to ·OH depends on the generation efficiency of H_2_O_2_ and the efficiency of the Fe^2+^ activation of H_2_O_2_. These mainly depend on the cathode catalyst. Therefore, the selection of electrode materials is the key component of the EF process, whose catalytic performance and service life directly affect the decomposition efficiency and treatment cost [12]. For example, by increasing the specific surface area, improving stability and oxidation–reduction reaction (ORR) catalytic activity, making it possible to generate H_2_O_2_ by ORR efficiently, or constructing a three-dimensional electrode EF, this system could achieve energy-saving and efficient operation [13,14,15,16]. It is also possible to improve treatment efficiency by coupling it with technologies such as electro-activated peroxydisulfate, bioelectrochemistry, and electro-adsorption [17,18,19] to expand applications.

Among the heterogeneous Fenton catalysts, zero-valent iron powder has better reduction ability and adsorption characteristics and is low-cost for pollutant treatment, but its application is limited due to the deactivation in catalyst activity and difficulty in collection, which causes secondary pollution [20,21,22,23]. However, the introduction of Fe MG has changed this situation. There are more active sites due to the unique amorphous structure and high specific surface area, enhancing the stability and activity of the catalysts. Moreover, Fe-based metallic glasses can optimize their catalytic performance by regulating their composition, making the Fenton degradation technology of Fe MGs highly adaptable in different conditions [24,25,26,27,28]. In addition, Fe MGs also have excellent electrocatalytic performance, controllable pore structure, and high conductivity with lower energy consumption and operating costs, making them equally advantageous in the EF process [29,30,31]. Researchers [32] applied Fe MG ribbons to EF catalysts for the first time, which greatly improved the degradation efficiency and provided a new method for the application of Fe MGs in the field of electrochemical degradation. However, there is less research on the mechanisms of EF process degradation technologies for Fe MGs, and their mechanism and optimization methods remain to be further explored.

Herein, an Fe_78_Si_9_B_13_ MG ribbon as the working electrode in a three-electrode system was constructed to degrade p-NP. The effect of voltage on the electrocatalytic efficiency and the free radicals produced in the degradation process of the catalyst were analyzed systematically. The results showed that when Fe_78_Si_9_B_13_ MG ribbons were used as cathodes, H_2_O_2_ could be produced in situ; therefore, ·OH could be further generated, which significantly improved the degradation efficiency of p-NP.

## 2. Experimental Design

### 2.1. Materials

Fe_78_B_22_ (wt%, 99.9% purity), Si (99.9% purity), and Fe (99.9% purity) were purchased from China New Metal Materials Technology Co., Ltd. (Beijing, China). NaOH and H_2_SO_4_ solutions (AR) were purchased from Sinopharm Chemical Reagent Co., Ltd. (Shanghai, China). Anhydrous sodium sulfate (Na_2_SO_4_, AR) and p-nitrophenol (p-NP, GC) were obtained from Xilong Scientific Co., Ltd. (Shantou, China) and Aladdin Chemical Regent Co., Ltd. (Shanghai, China), respectively. The specific concentration solutions needed for the experiment were dissolved in deionized water.

### 2.2. Preparation of Fe_78_Si_9_B_13_ MG Ribbons

The alloy ingot with Fe_78_Si_9_B_13_ (at%) was prepared in an arc-melting furnace (VF-AMP30, MAKABE Corporation, Tokyo, Japan). Fe_78_Si_9_B_13_ MG ribbons of about 1 mm in width and 15 μm in thickness were prepared by melt-spinning in a liquid metal supercooling device at about 1253 K (VF-RQT50, MAKABE, Japan). These ribbons were cut into 4 cm pieces for subsequent degradation. The structure of the ribbons was proven through an X-ray diffractometer (SmartLab, Rigaku, Akishima, Japan) using the Kα (Cu, 0.15418 nm).

### 2.3. Electro-Fenton Experiments

**Figure 1:**
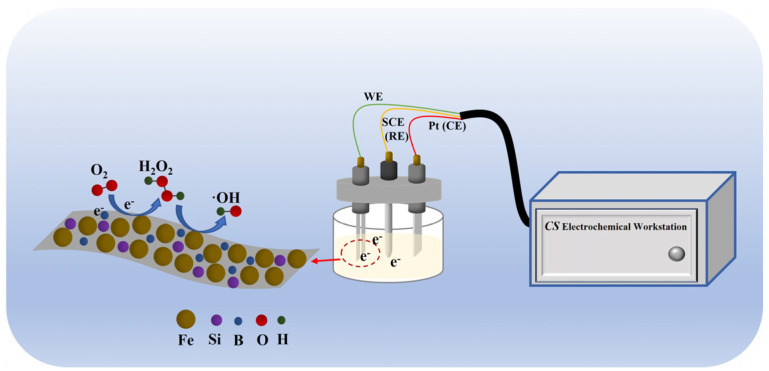
Schematical diagram of the experiments.

The EF process is an advanced oxidation method used for degrading organic pollutants in water. Its basic principle involves generating H_2_O_2_ electrochemically at the cathode by reducing oxygen (Equation (1)). Simultaneously, Fe^2^⁺ is introduced, which reacts with H_2_O_2_ to produce ·OH via the Fenton reaction (Equation (2)). This highly reactive ·OH then oxidizes and breaks down organic pollutants into smaller, less harmful compounds. Here, a three-electrode setup consisting of Fe_78_Si_9_B_13_ MG ribbons (4 cm × 0.8 cm), platinum sheets (1.5 cm × 2 cm), and SCE as the working, counter, and reference electrodes, respectively, was used to carry out the electro-Fenton reactions. The spacing between each electrode in this three-electrode system was 1 cm. The 8 mm wide ribbons were selected to ensure complete contact with the platinum electrode holder and consistent testing results. Na_2_SO_4_ (0.05 mol·L^−1^) was used as the electrolyte to enhance the conductivity of the solution. The constant voltage was provided by an electrochemical workstation (CS350, CORRTEST, Wuhan, China). Twenty-five milliliters of p-NP solution (20 mg/L) was prepared, and the pH was adjusted to 3 with H_2_SO_4_ and NaOH. The magnetic stirring speed was set to 500 rad/min to ensure sufficient reaction during the degradation process. The chemical degradation of p-NP used Fe_78_Si_9_B_13_ MG ribbons under open-circuit conditions without any current. The Fe_78_Si_9_B_13_ MG ribbons were repeatedly used for p-NP degradation without any treatment to investigate their cycling stability. The schematical diagram of the experiments is shown in Figure 1.

O_2_ + 2H^+^ + 2e^−^→H_2_O_2_ (1)

Fe^2+^ + H_2_O_2_→ ≡ Fe^3+^ + ⋅OH + OH^−^ (2)

### 2.4. Material Characterization

Structural analysis of the prepared ribbon was performed using an X-ray diffractometer (XRD, Smart lab, Rigaku, Japan). After the experiment, the ribbon was cleaned using ethanol and ultrasound to remove residues on the surface. Then, the samples were dried and further analyzed using XRD and scanning electron microscopy (SEM, SU-70, Hitachi, Chiyoda, Japan) to investigate structural changes and the microstructure before and after the reaction.

### 2.5. Analytical Methods

The relative concentration of ·OH was determined by photoluminescence spectroscopy (PL, FL970, Tiangen, Shanghai, China), following the steps outlined in the supporting information. One milliliter of the degradation solution was taken every certain interval and injected into a centrifuge tube containing 50 μL of NaOH (5 mol·L^−1^) and 2 mL of water. The residual p-NP concentration in the solution was determined through ultraviolet spectrophotometer testing at 400 nm after centrifugal filtration of the mixed solution. The formula of the degradation rate is shown in Equation (3).

D = (C_0_ − C_t_)/C_0_ × 100% (3)

where C_0_ and C_t_ are the initial concentration and the instantaneous concentration at reaction time t, respectively. D presents the degradation rate. Unless otherwise noted, all experiments were repeated three times to enhance the reliability of the experimental results.

## 3. Results and Discussion

### 3.1. Characterization

**Figure 2:**
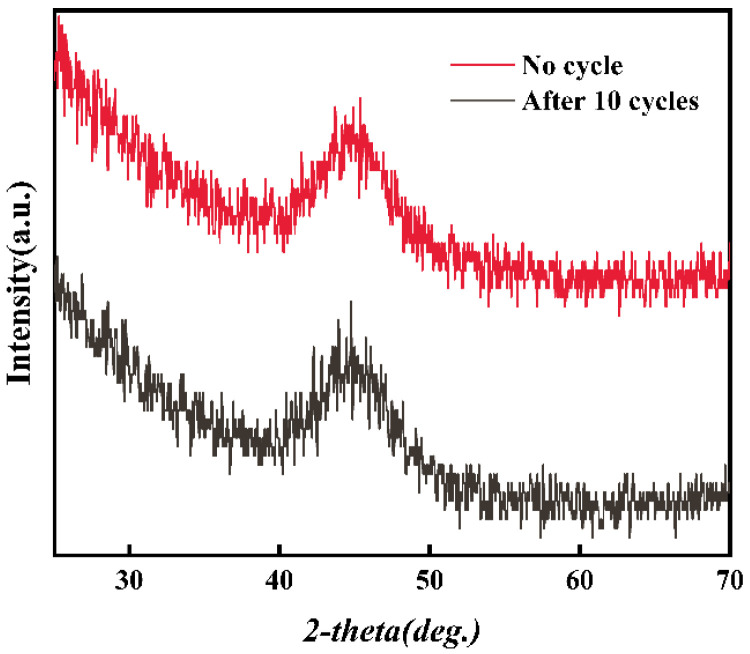
XRD patterns of the Fe_78_Si_9_B_13_ MG ribbons before and after 10 cycles.

As shown in Figure 2, the Fe_78_Si_9_B_13_ MG ribbons had a completely amorphous structure within the accuracy range of the XRD because the XRD analysis showed a wide diffuse scattering hump between 40° and 50° and no sharp peaks, representing the crystalline phase. Also, we could not find any obvious fracture or depression on the surfaces of as-prepared ribbons.

### 3.2. Effect of Electrode Voltage on Degradation Efficiency

**Figure 3:**
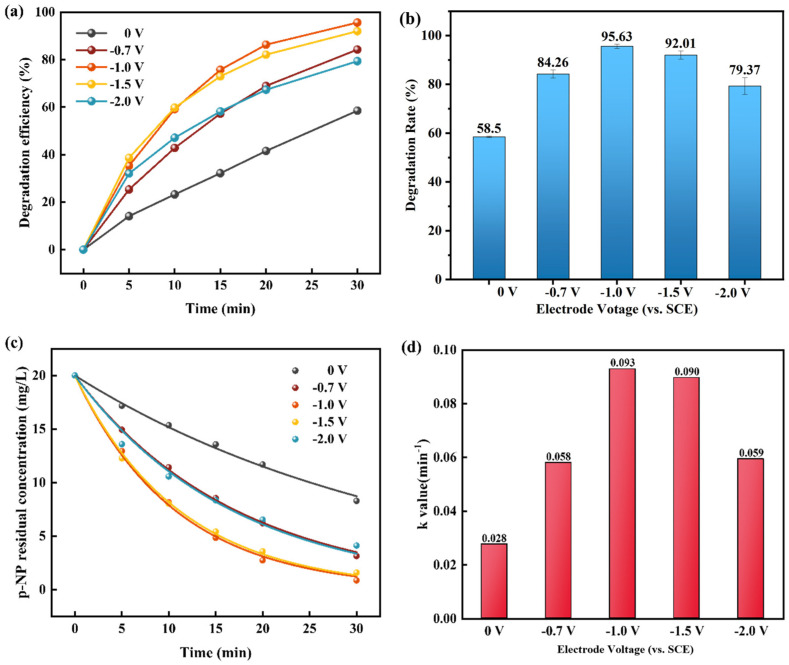
The degradation rate dependence of electrode voltage. (**a**) The removal efficiency of p-NP at different voltages. (**b**) The final removal ratio of p-NP at different voltages in 30 min. (**c**) The degradation kinetics curves of p-NP. (**d**) Reaction kinetics constant *k* of degradation reaction with different voltages.

Voltage is an important parameter in the electro-Fenton process [33]. The voltage range was set to −0.7 V~−2.0 V to determine the degradation performance with different voltages. Figure 3 shows the degradation rate of the Fe_78_Si_9_B_13_ MG ribbon cathode electro-Fenton process with different voltages. The Fe_78_Si_9_B_13_ MG ribbons with high catalytic activity exhibited excellent cathodic electro-Fenton process degradation performance. With increasing voltage, the degradation rate of p-NP by the Fe_78_Si_9_B_13_ MG ribbon showed a trend of increasing first and then decreasing after 30 min, as shown in Figure 3a. When the voltage was increased from −0.7 V to −1 V, the degradation rate of p-NP reached its peak because the generation of H_2_O_2_ at the cathode was affected by the voltage. H_2_O_2_ was almost impossible to generate at lower voltage, and the concentration of dissolved Fe^2+^ in the system was too small to maintain the efficient operation of the EF process. The theoretical hydrolysis voltage was 1.23 V. When the voltage was lower than 1.23 V, the O_2_ needed for the electric Fenton process mainly came from the air (the electrolysis cell was in the open state). When the voltage was higher than the theoretical voltage, the oxygen produced by electrolysis of water was near the anode. Therefore, water electrolysis occurred when the voltage was increased, and the decomposition of H_2_O_2_ was accelerated, causing Fe^2+^ to oxidize to Fe^3+^ with poor reactivity prematurely, resulting in different sizes of ferric hydroxide flocs that hindered the reaction, leading to a decrease in degradation efficiency [34].

To facilitate a comparison of degradation rates with different voltages, the residual concentration curve after the degradation reaction was fit with the first-order kinetics residual concentration fitting formula (Equation (4)):

C_t_ = C_0_ × e ^−kt^ (4)

where C_0_ and C_t_ are the initial concentration and instantaneous concentration by fitting at reaction time t, respectively, and k is the degradation reaction rate constant. The fitting curve is shown in Figure 3c and Table 1 contains the k values of p-NP degradation obtained by fitting.

It can be seen from Table 1 that a high ultimate degradation rate above 79% in 30 min for different applied voltages was obtained, which was superior to no voltage being applied. Among the voltages, −0.1 V was the best and ensured a 95.63% ultimate degradation rate in 30 min. Compared with the traditional AOP method [27], the EF process in this work exhibited higher degradation efficiency and also avoided the addition of extra hydrogen peroxide. Among the EF processes [8,35,36,37,38,39], the degradation efficiency in this work had a higher value or shorter reaction time and a comparable value with some modified new materials [39]. The compared results can be found in Table 2.

### 3.3. Mechanism of the Electro-Fenton Process

**Figure 4:**
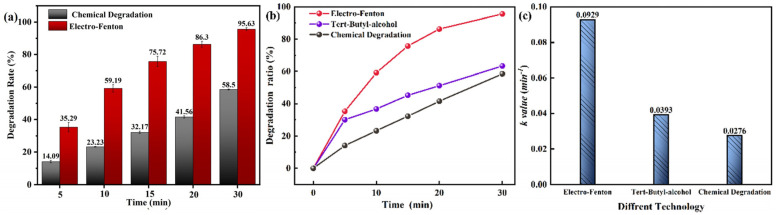
(**a**) D_t_ of the Fe_92.4_Si_4.8_B_2.8_ ribbon in different systems. (**b**) D_t_ of the Fe_78_Si_9_B_13_ MG ribbon in different systems. (**c**) *k* of the Fe_78_Si_9_B_13_ MG ribbon in different systems.

It is well known that the degradation rate of Fe-Si-B MG ribbons is positively correlated with the Boron content [40], and the degradation efficiency of Fe MG ribbons is better than that of crystalline ribbons in Fenton-like systems [41]. The crystalline Fe_92.4_Si_4.8_B_2.8_ ribbon was prepared for degradation. Figure 4a shows that the D of the electro-Fenton process with the ribbon was 95% at 30 min, which was 43.73% higher than that of chemical degradation. In contrast, the electro-Fenton degradation rate of the Fe_78_Si_9_B_13_ MG ribbon was also 95.63%, but its chemical degradation efficiency was higher than that of the crystalline Fe_92.4_Si_4.8_B_2.8_ ribbon. This indicated that the electro-Fenton degradation efficiency of the Fe-Si-B MG ribbon was insensitive to Boron content.

Tert-butyl alcohol (TBA) is a typical ·OH quencher, and the degradation of p-NP could be inhibited with high concentrations of TBA [42]. To explain the degradation mechanism of the Fe_78_Si_9_B_13_ MG ribbon cathode and the role of ·OH in the EF process, we designed quenching experiments to measure the concentrations of p-NP under different voltages. As shown in Figure 4b, the degradation rate of p-NP by the Fe_78_Si_9_B_13_ MG ribbon reached 95.63% in the EF process, the degradation rate decreased to 63.32% after the addition of TBA, and the rate was only 58.5% in 30 min with open circuit conditions, indicating the presence of ·OH in the electro-Fenton process. The synergistic effect of chemical degradation and electrochemical processes such as anodic oxidation resulted in degradation rates of 58.5% and 4.82% for p-NP, respectively.

**Figure 5:**
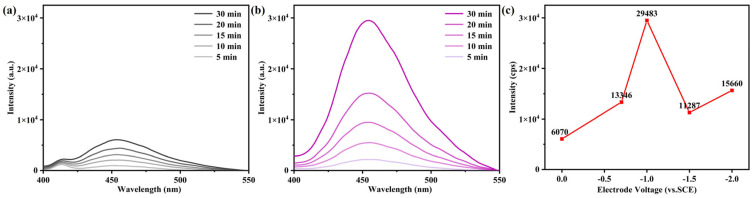
The results of the ·OH detection experiment. (**a**) Chemical degradation. (**b**) Electro-Fenton process. (**c**) ·OH production dependence of voltage at 30 min.

Coumarin was used as a probe, and a fluorescence method was used to test ·OH to investigate the impact of ·OH yield on the degradation efficiency of p-NP. As shown in Figure 5a,b, the PL signal appeared at 455 nm, which represented 7-hydroxycoumarin (7-HC). The PL signal of 7-HC in the Fe_78_Si_9_B_13_ MG ribbon electro-Fenton process was 4.86 times that of the chemical process. Since the intensity of the PL signal was positively correlated with the concentration of ·OH, this indicated that the electro-Fenton process produced a higher content of ·OH. Figure 5c shows the ·OH production dependence of the voltage at 30 min, which indicated that the optimal (corresponding to the largest PL signal of 7-HC) voltage was −1 V (vs. SCE). The generation rate of p-NP at different voltages was consistent with the degradation efficiency, shown in Figure 3a.

Based on the above results, a possible degradation pathway could be inferred for the Fe_78_Si_9_B_13_ MG ribbon cathode in the electro-Fenton process: First, O_2_ that naturally diffused to the Fe_78_Si_9_B_13_ MG ribbon interface accepted two electrons from the electrolytic cell to form H_2_O_2_ (Equation (1)). Subsequently, the H_2_O_2_ generated was activated by Fe^2+^ on the surface of the Fe_78_Si_9_B_13_ MG ribbon to form ⋅OH, with Fe^2+^ oxidized to Fe^3+^ (Equation (2)) [42].

### 3.4. Stability Assessment of Fe_78_Si_9_B_13_ MG

**Figure 6:**
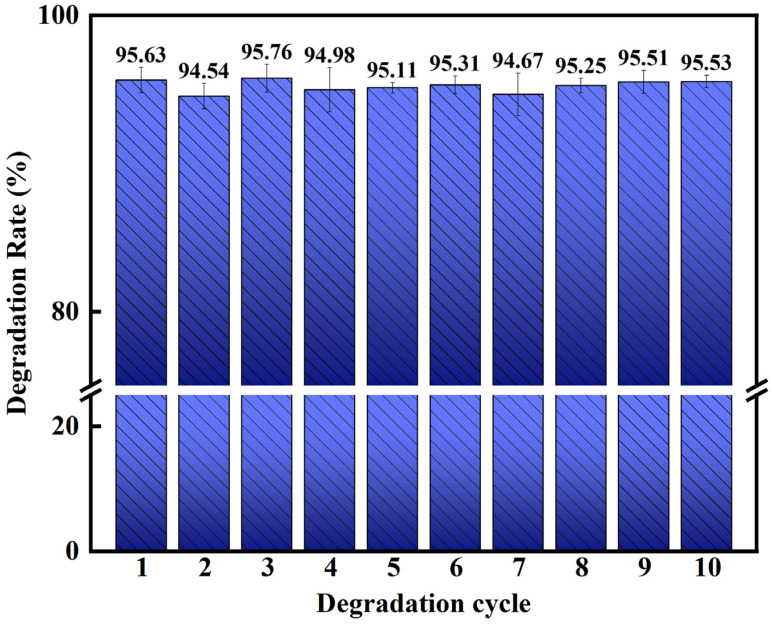
Degradation efficiency of different cycles for Fe_78_Si_9_B_13_ MG ribbons.

Figure 6 shows that the reusability and catalytic stability of the Fe_78_Si_9_B_13_ MG for the degradation of p-NP were confirmed in the cycle tests. The k and D of p-NP at 30 min were similar to the first performance. The ribbon still possessed a completely amorphous structure after 10 cycles, as shown in Figure 1, which benefited from the Si and B alloying, enhancing the amorphous formation and antioxidative capability and promoting the degradation reaction [43,44,45].

**Figure 7:**
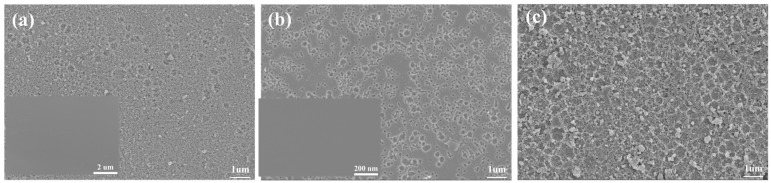
Morphology of the Fe_78_Si_9_B_13_ MG ribbons after use. (**a**) After one chemical degradation (**b**) after one EF process and (**c**) after 10 cycles EF processes, inset with the corresponding surface morphology of the as-prepared ribbons.

Figure 7 shows SEM micrographs of the Fe_78_Si_9_B_13_ MG after electrocatalytic and chemical degradation, inset with the corresponding surface morphology of the as-prepared ribbons. The surfaces on the ribbons presented iron oxides or hydroxides (such as Fe_2_O_3_, FeOOH, Fe(OH)_2_, and Fe(OH)_3_) formed by the oxidation of iron during the chemical degradation process to hinder further reactions. However, the surface of Fe_78_Si_9_B_13_ resulted in fine spots (approximately 500 nm) spalling under the action of the current. Fresh Fe^0^ was continuously exposed and participated in degradation without oxide, so it could be reused without treatment.

## 4. Conclusions

In this work, Fe_78_Si_9_B_13_ MG ribbons were used as cathodes and showed excellent degradation performance for p-NP. When the electrode voltage was −1 V (vs. SCE), the degradation efficiency was the best, with a degradation rate of 95.63% in 30 min, and the reaction kinetics constant *k* was 0.093, which was 3.4 times that of chemical degradation. The PL analysis indicated that the concentration of ·OH in the electro-Fenton system was 4.86 times higher than that of chemical degradation, and more ·OH generated was conducive to enhancing the degradation efficiency. In addition, the Fe_78_Si_9_B_13_ MG ribbon exhibited good catalytic stability. It could be reused at least 10 times without apparent catalytic efficiency loss. The degradation rate for each cycle exceeded 93% and it still maintained a complete amorphous structure even after multiple uses. This will accelerate the application of Fe-based MGs in wastewater treatment.

## Figures and Tables

**Table 1:** The EF degradation efficiency of p-NP at different voltages using the Fe_78_Si_9_B_13_ MG ribbon in 30 min.

Voltage (vs. SCE)	C_ultimate_ (mg·L^−1^)	D_ultimate_ (%)	k (min^−1^)	R^2^
0 V	8.300	58.50	0.028	0.97
−0.7 V	3.148	84.26	0.058	0.99
−1.0 V	0.874	95.63	0.093	0.99
−1.5 V	1.598	92.01	0.089	0.99
−2.0 V	4.126	79.37	0.059	0.98

**Table 2:** The degradation efficiency of organic pollutants using the Fe_78_Si_9_B_13_ MG ribbon (EF process) compared with references.

Material	Voltage/Current	Pollutants	Degradation Rate (%)	Ref.
Fe_78_Si_9_B_13_	AOP	RhB (20 mg/L)	90% (60 min)	Ref. [27]
Carbon	−0.6 V	PFOA (10 mg/L)	92.3–98.0% (2–3 h)	Ref. [8]
Fe	20 V	Acid red 18 dye (100 mg/L)	85.5% (15 min)	Ref. [35]
Fe_78_Si_9_B_13_	6.5 mA/cm^2^	Petroleum Wastewater	COD 89.6% (100 min)	Ref. [36]
Cu_55_Zr_45_	50 mA	AOII (50 mg/L)	95.6% (40 min)	Ref. [37]
FeSiBNbCu	−0.5 V	Orange II (20 mg/L)	Around 40% (60 min)	Ref. [38]
FeSiBNbCu @Cu2.56			Exceeded 90% (60 min)	
FC12-NC@CF	6 mA/cm^2^	Norfloxacin (20 mg/L)	98.64% (90 min)	Ref. [39]
CF			56.19% (90 min)	
Fe_78_Si_9_B_13_	−1.0 V	p-NP (20 mg/L)	95.63% (30 min)	This work

## Data Availability

The original contributions presented in this study are included in the article. Further inquiries can be directed to the corresponding author.
